# Observation of reentrant metal-insulator transition in a random-dimer disordered SSH lattice

**DOI:** 10.1038/s44310-024-00008-7

**Published:** 2024-06-03

**Authors:** Ze-Sheng Xu, Jun Gao, Adrian Iovan, Ivan M. Khaymovich, Val Zwiller, Ali W. Elshaari

**Affiliations:** 1 Department of Applied Physics, KTH Royal Institute of Technology, Albanova University Centre, Roslagstullsbacken 21, 106 91 Stockholm, Sweden; 2 Nordita, Stockholm University and KTH Royal Institute of Technology, Hannes Alfvéns väg 12, SE-106 91 Stockholm, Sweden; 3 Institute for Physics of Microstructures, Russian Academy of Sciences, 603950 Nizhny, Novgorod, GSP-105 Russia

**Keywords:** Silicon photonics, Optics and photonics, Integrated optics

## Abstract

The interrelationship between localization, quantum transport, and disorder has remained a fascinating focus in scientific research. Traditionally, it has been widely accepted in the physics community that in one-dimensional systems, as disorder increases, localization intensifies, triggering a metal-insulator transition. However, a recent theoretical investigation [Phys. Rev. Lett. 126, 106803] has revealed that the interplay between dimerization and disorder leads to a reentrant localization transition, constituting a remarkable theoretical advancement in the field. Here, we present the first experimental observation of reentrant localization using an experimentally friendly model, a photonic SSH lattice with random-dimer disorder, achieved by incrementally adjusting synthetic potentials. In the presence of correlated on-site potentials, certain eigenstates exhibit extended behavior following the localization transition as the disorder continues to increase. We directly probe the wave function in disordered lattices by exciting specific lattice sites and recording the light distribution. This reentrant phenomenon is further verified by observing an anomalous peak in the normalized participation ratio. Our study enriches the understanding of transport in disordered mediums and accentuates the substantial potential of integrated photonics for the simulation of intricate condensed matter physics phenomena.

## Introduction

Since the pioneering work of Rolf Landauer and Philip. W. Anderson^1,2^, the phenomenon of wave function localization in disordered media has been extensively investigated, exploring the quantum phase transition and the transport physics in disordered systems. Anderson localization predicts the absence of diffusive behavior in the single-particle wave function when subjected to a disorder potential, arising from wave interference effects between multiple propagation paths. As the disorder strength increases, transport becomes suppressed resulting in the metal-insulator transition. Extensive studies have been conducted on this transition in diverse systems, including electron gases, doped semiconductors, random media, photonic lattices, ultra-cold optical lattices, ultrasound, and atomic system^3–11^. The prior work on low-dimensional systems (*D* ≤ 2) establishes that uncorrelated disorder can shift all eigenstates to a localized phase, regardless of the disorder amplitude^12^. In contrast, a quasiperiodic potential reveals that a non-zero critical disorder strength can differentiate the metallic and insulator phases, generating an intermediate region where both extended and localized states coexist^13–18^. This intermediate region is defined by the single-particle mobility edge (at least at finite system sizes)^4,19–25^, symbolizing a critical energy threshold distinguishing the boundary between the extended and localized states in the system (In some short-range uncorrelated disordered models, the coexistence of extended and localized states also appears and do not show the mobility edge formation, see, e.g.,^26^).

Historically, the prevailing belief held that the system would undergo a unidirectional transition from the extended state to the localized state as the disorder increases. Contrary to this understanding, recent studies illustrate that the reentrant localization can be achieved in a Su-Schrieffer-Heeger (SSH) chain with staggered quasiperiodic disorder^27,28^ or random-dimer disorder^29^. This implies that with a further increase in disorder, two transitions ensue, leading to a regime with the coexistence of extended and localized states and further to the second localized phase, respectively. The genesis of this phenomenon resides in the increase of the disorder strength, which results in the re-extension of some previously localized states. Next, at even higher disorder strength, the above-delocalized states tend to the localization again. This shows a strong correlation with the coexistence of extended and localized states in different spectral parts.

In this work, we implement a correlated disorder by imposing a specific potential on the random dimer of an SSH chain. To achieve this, we employ a Si_3_N_4_ waveguide array fabricated using electron beam lithography and reactive ion etching^30–32^, facilitating effective control over the nearest-neighbor hopping amplitude. In addition, we tailor the width of array waveguides to introduce a synthetic on-site potential^33,34^. To examine the system, we selectively excite a specific single site within the waveguide array. Subsequently, we adopt a top imaging strategy to probe the intensity distribution at the end of the array by analyzing the scattered light. These images are recorded for different disorder strengths, and the so-called normalized participation ratios (NPR), characterizing the localization-delocalization phase diagram, are extracted. In addition to observing light diffusion or localized behavior corresponding to different quantum transport regimes, we also discern a consistent trend in the NPR of single-site excitation that aligns with our theoretical model. The concordance between experimental observations and theoretical predictions provides compelling evidence of the non-monotonic metal-insulator transition in this one-dimensional photonic system. This reentrant localization transition, observed experimentally, opens a promising pathway for studying condensed matter physics within an on-chip nano-photonic system. Additionally, the property of modulating the wave-packet distribution in the lattice be potentially explored for encoding quantum states on chip^35–38^.

## Results

Here, we consider a one-dimensional SSH model with random-dimer disorder, which is described by the following Hamiltonian

1 $$H=t\mathop{\sum }\limits_{n=1}^{N-1}\left[1-{(-1)}^{n}\Delta \right]\left({c}_{n}^{{\dagger} }{c}_{n+1}+\,{{\mbox{H}}}.{{\mbox{c}}}\,.\right)+\mathop{\sum }\limits_{n=1}^{N/2}{\epsilon }_{n}({c}_{2n-1}^{{\dagger} }{c}_{2n-1}+{c}_{2n}^{{\dagger} }{c}_{2n}),$$

2 $${\epsilon }_{n}=\left\{\begin{array}{ll}\epsilon ,\quad \,{{\mbox{probability}}}\,=p\\ 0,\quad \,{{\mbox{probability}}}\,=1-p\end{array}\right.$$

This model comprises *N*/2 unit cells, each containing two sites, where ${c}_{n}^{{\dagger} }$ and *c*_*n*_ represent the creation and annihilation operators corresponding to the site *n*. The nearest-neighbor hopping terms *t*(1 ± Δ), with the dimerization parameter Δ, represent the intra-cell and inter-cell coupling strength between sites, respectively. *ϵ*_*n*_ designates the bimodally distributed on-site potential applied to the random dimer, being *ϵ* with the probability *p* and zero otherwise. By tuning the value of *ϵ*, we can probe the different transport regimes occurring at different disorder strengths. This model presents the reentrant localization at the specific critical values of *ϵ*, which can be examined by analyzing the NPR in conjunction with the inverse participation ratio (IPR)^21,39^. The definitions of these metrics are provided as follows:

3 $${{{{\rm{IPR}}}}}_{n}=\mathop{\sum }\limits_{i=1}^{N}{\left\vert {\phi }_{n}^{i}\right\vert }^{4}\,,\quad {{{{\rm{NPR}}}}}_{n}=\frac{1}{{{{\rm{N}}}}* {{{\rm{IPR}}}}}={\left(N\mathop{\sum }\limits_{i = 1}^{N}{\left\vert {\phi }_{n}^{i}\right\vert }^{4}\right)}^{-1},$$

where ${\phi }_{n}^{i}$ denotes the *n*-th eigenstate coefficient at the site 1 ≤ *i* ≤ *N*, while *N* signifies the number of sites in the lattice. It is theoretically proposed that in the thermodynamic limit, as *N* tends towards infinity, the NPR stays finite when the IPR vanishes for an ergodically extended state. Conversely, the NPR approaches zero when the IPR stays finite for a localized state. Further, we will use both energy-resolved and mean values across the spectrum of the above two measures, defined as follows^40^

4 $$\langle {{{\rm{NPR}}}}\rangle =\frac{1}{N}\mathop{\sum }\limits_{i=1}^{N}{{{{\rm{NPR}}}}}_{n},\quad \langle {{{\rm{IPR}}}}\rangle =\frac{1}{N}\mathop{\sum }\limits_{i=1}^{N}{{{{\rm{IPR}}}}}_{n}.$$

When both the average values 〈NPR〉 and 〈IPR〉 are simultaneously finite, it suggests the coexistence of localized and extended states. This gives rise to the intermediate regime, triggering the phenomenon of reentrant localization and inducing the non-monotonic metal-insulator transition within the system. However, relying solely on the NPR or IPR values in our investigation poses difficulties in accurately identifying the coexistence of eigenstates within the extended and localized phases. One feasible approach is the simultaneous calculation of the NPR and IPR along the random-dimer onsite potential, followed by the analysis of the non-zero region for both these parameters. An alternative strategy involves computing a defined parameter *η* as $\eta ={\log }_{10}[\langle {{{\rm{IPR}}}}\rangle \times \langle {{{\rm{NPR}}}}\rangle ]$^39^. By assessing the ratio of extended to localized states within all *N* eigenstates, we establish the criteria for the regime where extended and localized states coexist (It is worth noting that the minimum value corresponds to at least one localized/extended state in the system). The detailed derivation can be found in the Supplementary information:

5 $${\log }_{{{{\rm{10}}}}}\left(\frac{2}{N}\right)\le \eta\, \le\, {\log }_{{{{\rm{10}}}}}\left(\frac{1}{4}+\frac{1}{2N}\right)$$

**Fig. 1:**
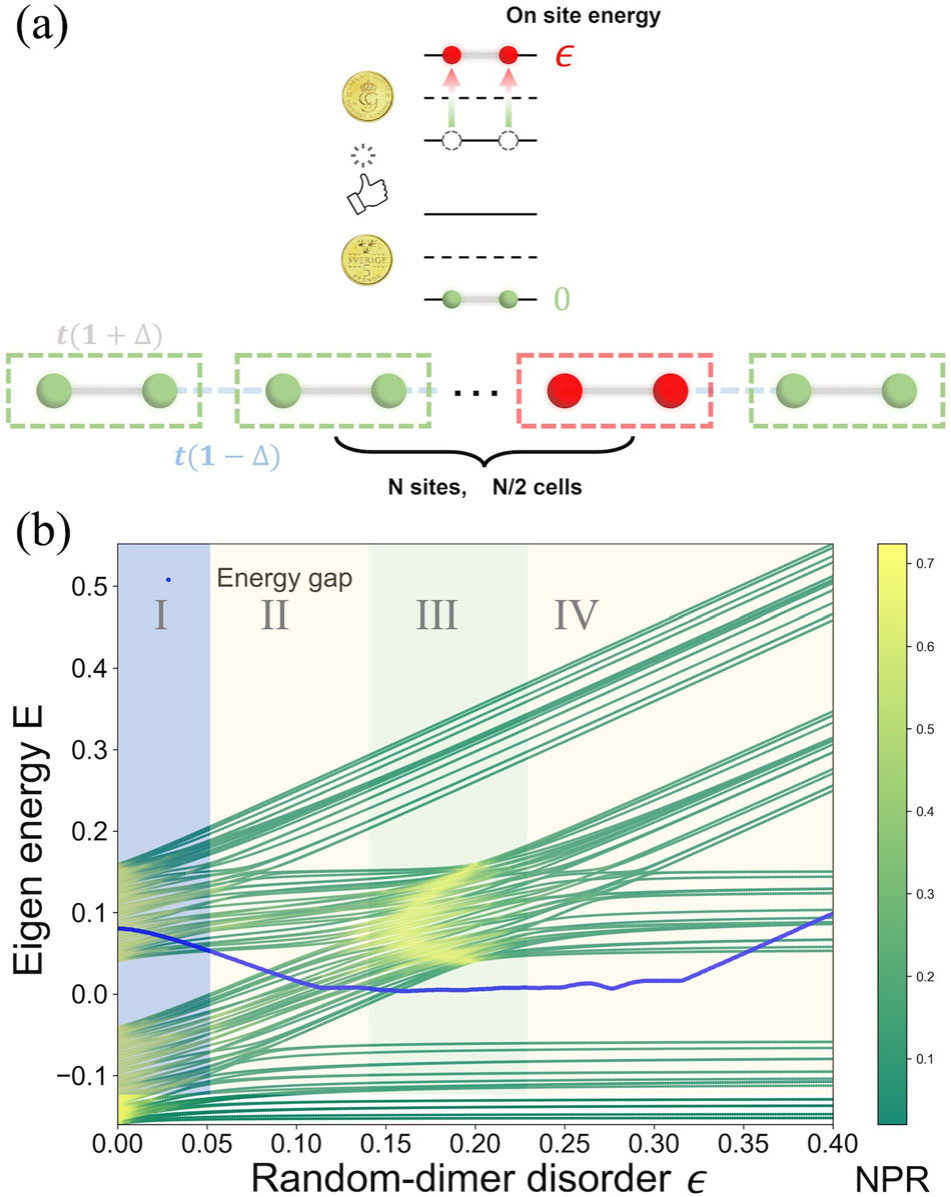
SSH Lattice with random-dimer disorder and dimerization amplitude Δ = 0.25 featuring an on-site potential and a hopping amplitude *t* = 0.08. **a** Schematic representation of the random-dimer disordered SSH model with model’s parameters. The two green nodes within the dotted box depict a situation devoid of on-site potential, whereas the two red nodes symbolize a dimer with a chosen finite on-site potential. The on-site potential is randomly allocated with a probability *p* = 0.5, analogous to the likelihood of getting heads in a fair coin toss. In a broad sense, our model’s on-site potential can be regarded as a bimodal telegraph noise. **b** Eigenenergy spectrum of the random-dimer disordered SSH model with varying on-site potential *ϵ*. The color from green to cyan indicates the NPR calculated with the eigenstates numerical method. I, II, III, and IV correspond to different regimes of disorder scales. The spectrum is clearly separated into four bands: energies of two of them increase linearly with *ϵ*, while those of the other two are insensitive to it. The corresponding wave functions of linear (constant) in *ϵ* live mostly at disordered *ϵ*_*n*_ = *ϵ* (clean *ϵ*_*n*_ = 0) dimers. In each pair of bands, these wave functions are also symmetric for the lower and antisymmetric for the higher one of the two bands, see Supplemental Information. As the disorder strength approaches two critical values, we observe the gap closure and reopening, indicative of the disappearing and reappearing in-gap edge states, associated with the non-monotonic insulator-metal-insulator transition. The blue curve represents the Energy gap in this system.

In our experiment, we select a lattice with *N* = 100 sites, as depicted in Fig. 1a, and generate a random-dimer number array with a probability *p* = 0.5 according to Eq. (2). We choose *t* = 0.08 and Δ = 0.25, so the intra/inter-cell coupling strength is 0.1 and 0.06, which sets the lattice at the topologically trivial phase^29^. Fig. 1b presents the results of sweeping the on-site potential from 0 to 0.4 across the random-dimer number array. Subsequently, we conduct numerical calculations to determine the lattice’s spectrum with 1000 sites, unveiling the system’s energy levels and their corresponding NPR. The spectrum of the system is given by four well-distinguished bands, centered around *E*_*ν*,±_ = *νϵ* ± *t*(1 + Δ), with wave functions, living mostly on the dimers with the on-site potential *ϵ*_*n*_ = *νϵ*, *ν* = 0, 1, and being symmetric (antisymmetric) at each of such dimers with the corresponding energy shift ± *t*(1 + Δ), respectively, see Supplemental Information. The width of each band is given by the smaller hopping 2*t*(1 − Δ). The blue curve in Fig. 1 uncovers the closing and reopening of the energy gap (the smallest energy difference within the middle band in the band-crossing region and the smallest energy difference between two middle subbands in the other region) in the system, signifying the behavior of wave packet. It is this gap closure which is responsible for the reentrant delocalization. Indeed, close to the intersection of 2 middle bands, at ∣*ϵ* − 2*t*(1 + Δ)∣ ≪ *t*, the effective disorder is given by the difference of energy-band centers *E*_*ν*,±_. As soon as 2 bands cross, this difference goes to zero and a disorder-free sector of the Hamiltonian with delocalized states emerges at this crossing-band pair. The states in the other pair of bands stay localized. This explains the reentrant character of the delocalization-localization phase diagram.

**Fig. 2:**
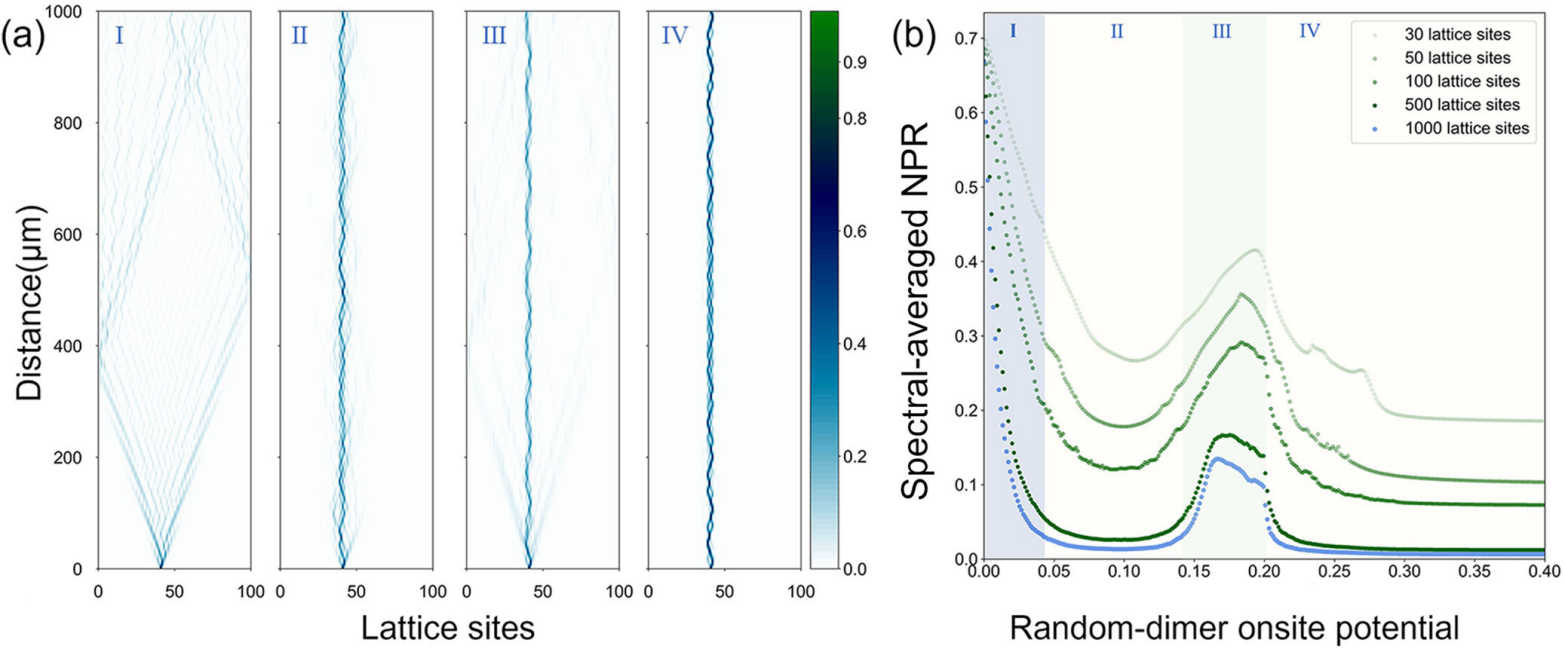
Light transport in the Lattice and Spectral-averaged NPR Under Varying Random-Dimer On-site Potential. **a** The four simulated trajectories of light inject from the 41st site and propagate in 1000*μ*m long SSH lattices comprising 100 sites with varying degrees of random dimer disorder are displayed. I, II, III, and IV correspond to random-dimer on-site potentials, *ϵ* = 0, 0.1, 0.185, and 0.3 respectively, with random-dimer assignment probability of *p* = 0.5. In I, a ballistic light propagation takes place without any disorder present; in II and IV, light becomes localized within the lattice at the designed random-dimer on-site potentials, despite significant propagation distance. III demonstrates the phenomenon of reentrant localization, where both extended and localized states coexist. **b** The numerically computed spectral-averaged NPR based on eigenstates as a function of random-dimer potential *ϵ* over a range of lattice sites from 30 to 1000. As the number of lattice sites increases, the spectral-averaged NPR exhibits a declining trend, yet the counterintuitive reentrant peaks consistently emerge. The four light transport simulations coincide with the different regimes in the spectral-averaged NPR plot, as denoted by the color coding and labeling I to IV.

Figure 2a displays the simulated pattern of the 41st single-site excitation obtained using the coupled mode theory^41–43^ to showcase the evolution of light within lattices having a length of 1000 *μ*m. The on-site potentials *ϵ* of these lattices are 0, 0.1, 0.185 and 0.3, from left to right. Comparing Fig. 2a III with II and I, we observe that the light undergoes a distinct behavior in III. In this intermediate regime III, a part of the light intensity stays near the initial location as in II, but another part ballistically spreads as in **I**. This gives evidence of a coexistence of localized and extended light, observed simultaneously. Unexpectedly this coexistence appears even after the light has transitioned into the fully localized regime **II** upon reaching the critical value of the on-site potential, *ϵ*. Furthermore, as we continuously increase *ϵ*, the light evolution pattern in regime IV reverts to exhibiting localized behavior, clearly demonstrating the occurrence of reentrant localization in this system. Fig. 2b shows the spectral-averaged NPR based on the eigenstates for the random-dimer on-site potential system with pre-defined parameters above while varying lattice sites numbers. In the limit of a large number of lattice sites, the 〈NPR〉 approaches its lower bound, which scales as $\sim \frac{1}{{{{\rm{N}}}}}$ (Although this value is often not reached because the most localized case in our model is that the wave is localized in a few sites rather than localized in one site). Despite the decreasing trend of the lower limits as the number of sites increases, the curves consistently exhibit an anomalous peak followed by a subsequent decrease. This observed behavior of the curves provides convincing evidence for the existence of reentrant localization in this system and justifies that the reentrant localization in our system with 100 sites does exist and can be experimentally observed.

**Fig. 3:**
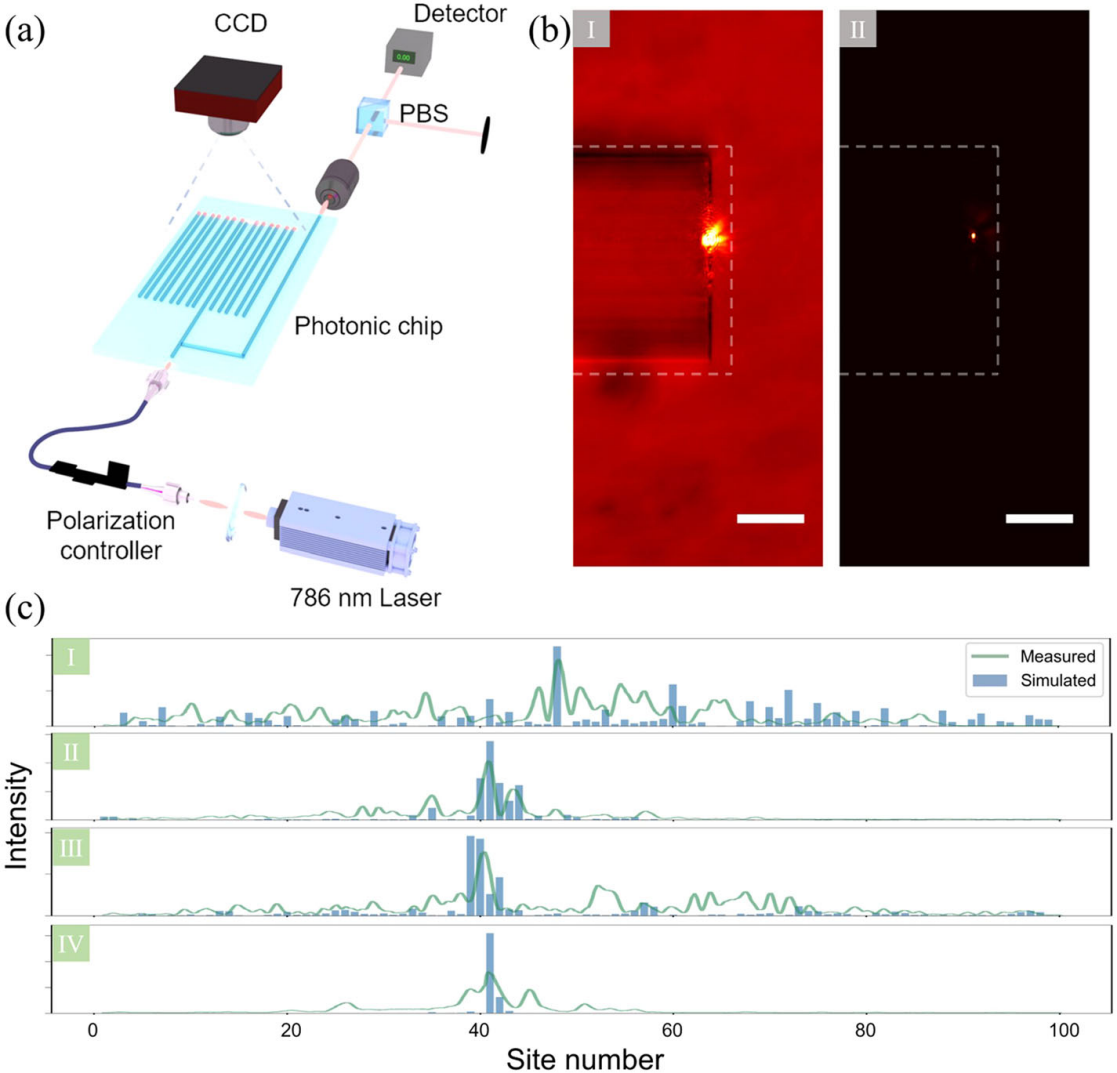
Details of the Experimental Setup and Collected Data. **a** Illustration of the experimental setup. A 786 nm continuous wave laser is utilized for the excitation of the random-dimer disordered SSH photonic lattice through a lensed fiber. A Y-splitter is incorporated to control polarization and optimize the coupling to the photonic chip. The Transverse Electric (TE) mode is excited by accurately adjusting the paddle of the polarization controller and monitoring the detector counts transmitted through the chip. A polarizing beam splitter enables exciting the TE modes by minimizing the detector count. The intensity of the facet light distribution is imaged using a microscope objective and a CCD camera. **b** Extraction of the output light distribution. The light propagates from the left to the right, and scatters at the end of this photonic lattice. Two images, both with identical focus, are captured to ascertain the distribution of light intensity. II represents the output pattern of the lattice, while I serves as a reference pattern of the illuminated lattice that aids in pinpointing exact coordinates in pixel space. The section within the dotted line in both images indicates the position of the lattice, obtained from the reference image. The position of each site is then extracted, and it is used to deduce the light intensity distribution in the lattice. The scale bar in I and II corresponds to the length of 20 *μ* m. **c** Comparison between experimentally measured and simulated light intensity distributions. I, II, III, and IV correspond to the output intensity distribution at a distance of 1000*μ*m, following the 41st single-site excitation with random-dimer on-site potentials *ϵ* = 0, 0.1, 0.185, and 0.3, respectively.

To investigate our system’s properties, we employ a full-vectorial mode solver^44^ for numerical simulations of the waveguide’s propagation constants. These propagation constants serve as a representation of the on-site potential within our model. Detailed analysis is presented in the Supplementary information. In our experiment, we utilized a CMOS-compatible Si_3_N_4_ photonic platform to successfully integrate nano-sized SSH devices with random-dimer disorder. Each device consists of *N* = 100 waveguides, with dimensions of 250 nm high and 450−518 nm wide according to the different on-site potentials, with SiO_2_ bottom and air top cladding. These waveguides support only the fundamental modes at a 786 nm wavelength. The width of and the gap between these waveguides are well-engineered to satisfy the designed hopping amplitude and the on-site potentials. The experimental setup is shown in Fig. 3a. We excite the random-disordered SSH lattice with a continuous-wave coherent laser centered at 786 nm. Using a lensed fiber fixed on a six-axis nano-positioning stage, the laser is finely coupled into the nanophotonic chip. A polarizing beam splitter with a three-paddle polarization controller is installed to selectively excite only the transverse electric (TE) mode. The on-chip Y-splitter divides the light into two paths, the first leading to the random-disordered SSH lattice, with the second acting as the monitor waveguide for tuning the coupling efficiency and controlling the polarization. The output pattern of the light intensity at the end of each lattice is collected from the scattered signal imaged with a 40X objective and captured by a charge-coupled device (CCD) camera. Fig. 3b presents captured images obtained under both non-illuminated and illuminated conditions, with the focus remaining consistent. By analyzing the region marked by dashed lines in Fig. 3b I, we extract the pixel coordinates corresponding to the photonic lattice structure. By combining this information with Fig. 3b II which depicts the output pattern of the lattice, we establish a relationship between the light intensity distribution and the lattice site numbers ranging from 1 to 100. This enables us to retrieve the NPR for the single-site excitation scenario, providing valuable insights into the system’s light behavior. Here the single-site excitation NPR is defined as the NPR, Eq. (3), for the wave-packet *ψ*^*i*^(*j*, *t*) at a certain evolution time *t*, which was initialized at *t* = 0 at a certain single site *j* (excited waveguide), *ψ*^*i*^(*j*, 0) = *δ*_*i*,*j*_, with *δ*_*i*,*j*_ being Kronecker delta:

6 $${{{\rm{IPR}}}}(j,t)=\mathop{\sum }\limits_{i=1}^{N}{\left\vert {\psi }^{i}(j,t)\right\vert }^{4}\,,\quad {{{\rm{NPR}}}}(j,t)=\frac{1}{{{{\rm{N}}}}* {{{\rm{IPR}}}}(j,t)}={\left(N\mathop{\sum }\limits_{i = 1}^{N}{\left\vert {\psi }^{i}(j,t)\right\vert }^{4}\right)}^{-1}\,,$$

Figure 3c shows the experimentally measured light distribution along the sites compared with the theoretically predicted light distribution. We can see the distinct signature of extended and localized wave packets transport in different regimes from I to IV. I indicates extended behavior with *ϵ* = 0, while II and IV demonstrate localized behavior. III represents the intermediate case. Specifically, in region II and IV the wave packet is highly confined within a few lattice sites, while in region **III** we see a clear signature that indicates the coexistence of extended and localized behavior. The peak and overall behavior of the measured light intensity distribution align well with the theoretical prediction. Notably, the laser light transport shows consistency with the eigenstates’ behavior, implying the non-monotonic metal-insulator transition and the reentrant localization.

**Fig. 4:**
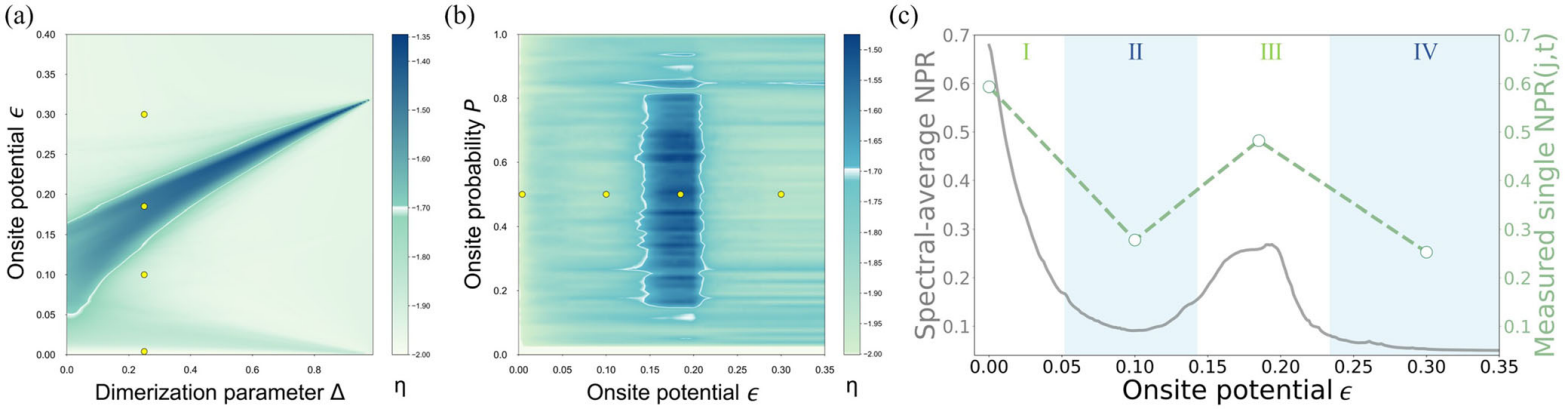
Phase diagram and retrieved NPR(*j*, *t*) for single-site excitation. **a** The phase diagram of *η* over the dimerization parameter Δ and on-site potential *ϵ* plane for a 100-site dimer-disordered SSH lattice. The blue region signifies the concurrent non-zero values of both 〈IPR〉 and 〈NPR〉, according to the definition $\eta ={\log }_{10}[\langle {{{\rm{IPR}}}}\rangle \times \langle {{{\rm{NPR}}}}\rangle ]$. Four small yellow circles in the diagram represent the experimental sampling points we selected where the dimerization parameter Δ = 0.25, corresponds to the four regions presented in Fig. 2b. The reentrant localization is validated by the coexistence of localized and extended states. **b** Phase diagram of *η* in the on-site potential *ϵ* and on-site probability p plane. The near-vertical border of the reentrant localization region accentuates that the critical values remain stable as the on-site probability varies widely (0.15 < *p* < 0.8), confirming that our choice of on-site probability *p* = 0.5 does not add any specificity. **c** The comparison between spectral-averaged NPR based on eigenstates and the single NPR(*j*, *t*) based on the single-site excitation. In this context, we consider the parameters wherein *j* is assigned a value of 41, signifying the occurrence of the 41st single-site excitation, and *t* is set to 1000, indicating a total propagation length of 1000 *μ* m. The trend of the spectral-average NPR results from the ensemble of statistical behaviors of all NPRs from single-site excitations. Specifically, the NPRs from single-site excitations which are linked with extended eigenstates contribute to the peak of the spectral-averaged NPR in the reentrant localization and trigger a non-monotonic metal-insulator transition in the one-dimensional random-dimer disordered SSH system.

To investigate the reentrant localization regime, we plot the phase diagram according to Eq. (5). Figure 4a reveals the *η* based on the eigenstates calculation over different dimerization parameters Δ and on-site potentials *ϵ*, while Fig. 4b shows the *η* based on the eigenstates calculation for different on-site potentials *ϵ* and on-site probabilities *p*. The white edges in these phase diagrams separate the localized/extended phases from the coexistence phase of both extended and localized states. Non-monotonic behavior with the on-site potential shows the reentrant localization which we are interested in. The four yellow circles mark the experimental sampling points for collecting the light distribution and calculating the single-site excitation NPR, Eq. (6). Figure 4c presents the NPR of single-site excitation compared with the simulated 〈NPR〉 based on the numerically calculated eigenstates. Four highlighted regions I, II, III, IV correspond to the four specific regimes depicted in Fig. 2, respectively. The measured NPR from the 41st single-site excitation contributes to the statistical behavior observed in the spectral-averaged 〈NPR〉 and exhibits a consistent trend, as depicted in the plot. The choice of the 41st site to be excited is not specific and for most of the sites the behavior is expected to be similar. The NPR function shows a non-monotonic trend, it decreases in value as the disorder strength increases, subsequently revealing an anomalous peak in the intermediate regime, and ultimately undergoing a secondary decrease for large disorder strength. Based on the experimental results, we unambiguously observe the occurrence of reentrant localization behavior in the random-dimer disordered SSH photonic lattices, both through intuitive visual inspection and analysis of NPR and IPR.

## Discussion

In conclusion, we have demonstrated the observation of the reentrant localization in a one-dimensional disordered system. This system is based on a nano-fabricated Si_3_N_4_ platform realized in photonic waveguide devices. The numerically calculated 〈NPR〉 and the phase diagram for the defined parameter *η* guided our choice of sampling points with different on-site potentials *ϵ* to illustrate the transitions. Our technique of fine-tuning the width of waveguides allows us to control the on-site potential for each dimer. By using a single-shot approach, which involves probing the light intensity at each lattice site using an edge-scattered structure, we can investigate and observe the transport of light within the system. This approach enables us to retrieve the normalized participation ratio (NPR) for the single-site excitation scenario. The counter-intuitive drop and increase of NPR indicate the occurrence of non-monotonic localization transitions, thus pointing to the presence of reentrant localization. Our work contributes to a deeper comprehension of the underlying mechanisms governing the behavior of quantum systems subjected to disorder. Such insights are invaluable in elucidating the complex nature of localization transitions and their implications in various physical systems. From a technical perspective, we incorporate a nanometer-level precision electron beam lithography process, resulting in a precise implementation of complex on-site potentials and hopping parameters. The field of micro-nano photonics, particularly with the inclusion of the reentrant localization, holds significant potential in various areas such as information processing, optical computing, hybrid photonic circuits, topological photonics, and photonic quantum technologies^45–49^.

### Supplementary information

Supplementary information

## Data Availability

The data generated in this study is available upon reasonable request from the corresponding author.
