# Supplementary material for: Observation of reentrant metal-insulator transition in a random-dimer disordered SSH lattice
Source: Npj Nanophoton. 2024 Jun 3;1(1):8. doi: 10.1038/s44310-024-00008-7 (PMC11159787; doi:10.1038/s44310-024-00008-7)
Supplement: Supplementary file 1 — Supplementary information [file 44310_2024_8_MOESM1_ESM.pdf]

Supplementary Material for
Observation of reentrant metal-insulator transition in a random-dimer disordered SSH lattice

Ze-Sheng Xu,[1,*] Jun Gao,[1,†] Adrian Iovan,[1] Ivan M. Khaymovich,[2,3] Val Zwiller,[1] and Ali W. Elshaari[1,‡]

[1]*Department of Applied Physics, KTH Royal Institute of Technology, Albanova University Centre, Roslagstullsbacken 21, 106 91 Stockholm, Sweden*
[2]*Nordita, Stockholm University and KTH Royal Institute of Technology, Hannes Alfvéns väg 12, SE-106 91 Stockholm, Sweden*
[3]*Institute for Physics of Microstructures, Russian Academy of Sciences, 603950 Nizhny Novgorod, GSP-105, Russia*

Appendix A: Analytical consideration of the range of the defined quantity η

In this section, we analytically discuss the definition and the range of the defined quantity η. We consider this quantity combined with our one-dimensional chain model.

This definition $\eta = \log_{10}[\langle\mathrm{IPR}\rangle \times \langle\mathrm{NPR}\rangle]$ is introduced to quantify the degree of the coexistence of localized and extended eigenstates in the certain disorder strength. The range of this quantity can be studied based on the ratio of localized/extended eigenstates and the corresponding NPR (the normalized participation ratio) and IPR (the inverse participation ratio). Here we list the definitions of the NPR and IPR:

$$\mathrm{NPR}_n = \left(N\sum_{i=1}^{N}\left|\phi_n^i\right|^4\right)^{-1}, \quad \mathrm{IPR}_n = \frac{1}{\mathrm{N}*\mathrm{NPR}} = \sum_{i=1}^{N}\left|\phi_n^i\right|^4. \tag{S1}$$

and the definitions of the averaged NPR and IPR

$$\langle\mathrm{NPR}\rangle = \frac{1}{N}\sum_{i=1}^{N}\mathrm{NPR}_n, \quad \langle\mathrm{IPR}\rangle = \frac{1}{N}\sum_{i=1}^{N}\mathrm{IPR}_n. \tag{S2}$$

When the site number of the chain is N (N is large enough), we first consider the values in extreme cases, when all the states are fully extended or localized and the single NPR_n and IPR_n are equal to each other and the average. For the fully extended eigenstates:

$$\langle\mathrm{NPR}\rangle = \mathrm{NPR}_n \sim \left(N*\sum_{i=1}^{N}\left(\frac{1}{N}\right)^2\right)^{-1} = 1, \quad \langle\mathrm{IPR}\rangle = \mathrm{IPR}_n \sim \sum_{i=1}^{N}\left(\frac{1}{N}\right)^2 = \frac{1}{N} \tag{S3}$$

For the fully localized eigenstates:

$$\langle\mathrm{NPR}\rangle = \mathrm{NPR}_n \sim (N*1)^{-1} = \frac{1}{N}, \quad \langle\mathrm{IPR}\rangle = \mathrm{IPR}_n \sim 1 \tag{S4}$$

If the system is in the reentrant regime, it means there exist both localized and extended eigenstates. The lowest limit (LL) of η is given by nearly fully localized/extended states with only one different state.

$$LL = \log_{10}[\langle\mathrm{IPR}\rangle \times \langle\mathrm{NPR}\rangle] = \log_{10}[\frac{1+(N-1)\frac{1}{N}}{N}] \sim \log_{10}\left[\frac{2}{N}\right] \tag{S5}$$

---

* zesheng@kth.se
† junga@kth.se
‡ elshaari@kth.se

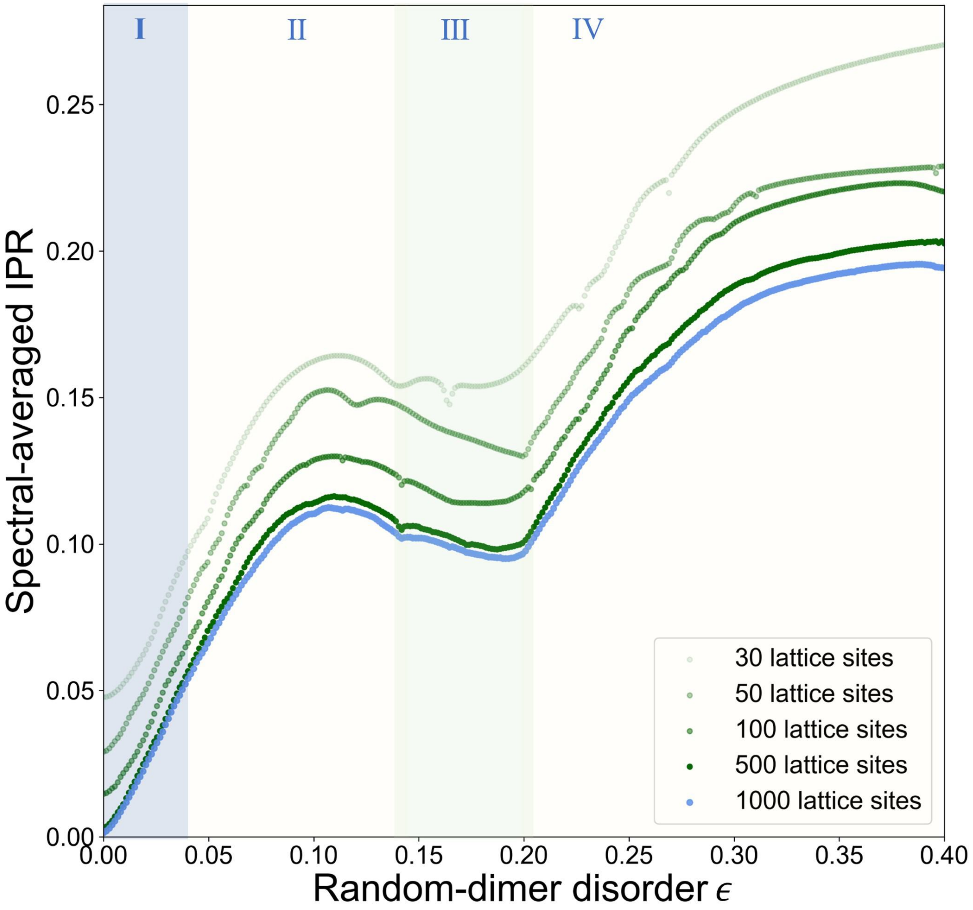

FIG. S1. Spectral-averaged IPR
The numerically computed spectral-averaged IPR of eigenstates as a function of random-dimer potential ϵ over a range of lattice sites from 30 to 1000. In Regime III, IPR shows an anomalous local minimum. As the number of sites increases, the value of IPR shows a downward trend and eventually converges when the number of sites becomes large enough.

For the maximum overlap case, we can assume that among N eigenstates X are extended, $N-X$ are localized, and the quantity η reaches its upper limit (UL):

$$UL = \log_{10}\left[\langle \mathrm{IPR}\rangle \times \langle \mathrm{NPR}\rangle\right] = \log_{10}\left[\frac{X * \frac{1}{N} + (N-X) * 1}{N} \times \frac{X * 1 + (N-X) * \frac{1}{N}}{N}\right] = \log_{10}\left[\frac{N-Y}{N} \times \frac{Y+1}{N}\right], \tag{S6}$$

with $Y = X(1-\frac{1}{N})$.

The maximum of the above expression for UL happens at the maximum of the quadratic function in brackets, i.e., at $Y = (N-1)/2$ or equivalently at $X = N/2$, leading to

$$UL = \log_{10}\left[\left(\frac{N+1}{2N}\right)^2\right] \simeq \log_{10}\left[\frac{1}{4} + \frac{1}{2N}\right] \tag{S7}$$

In summary, the range of η for reentrant localization region is:

$$\log_{10}\left[\frac{2}{N}\right] \leq \eta \leq \log_{10}\left[\frac{1}{4}+\frac{1}{2N}\right] \tag{S8}$$

In Fig. S1, we plot the spectral-averaged IPR for the experimentally realized setup with $p = 0.5$, $\Delta = 0.25$, $t = 0.08$ versus the amplitude of the disorder potential ϵ. Comparing this figure with Fig. 2(b) of the main text, one can see the slightly different effects of the spectral averaging: in IPR, Fig. S1, the presence of the finite fraction $1 - X/N$ of the localized states tends to its saturation with the system size, while NPR, Fig. 2(b) should go to a finite value for non-zero X/N. Instead, its tendency to decay with increasing system size hints us that X/N goes to zero, though the number of extended states in the regime III is still non-zero. This is also visible from Fig. 1(b) of the main text.

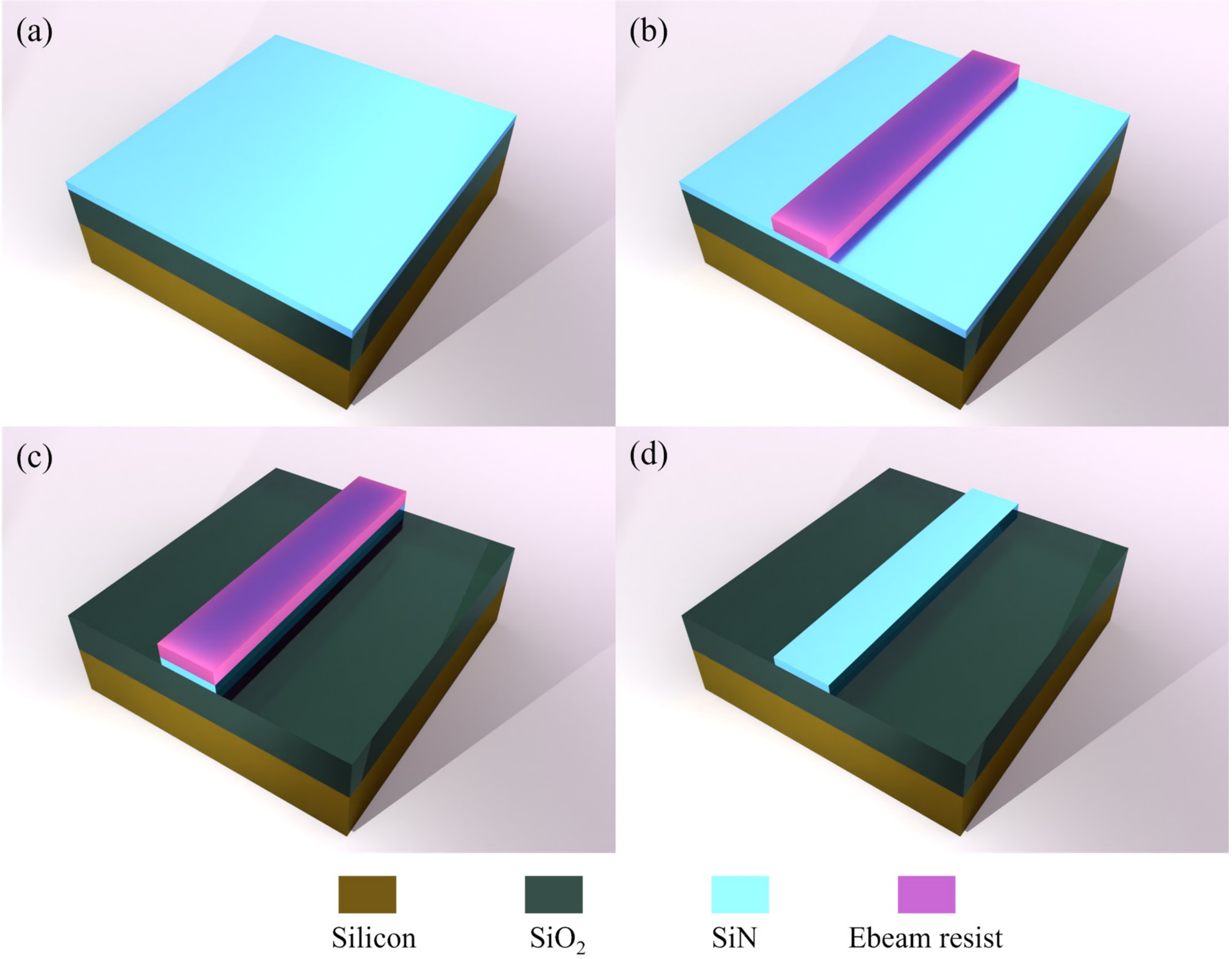

FIG. S2. Schematic of the nano-fabrication process
(a) SiO_2 and Si_3N_4 coating **(b)** Electron beam lithography and unexposed regions removing **(c)** Dry etching**(d)** Resist removing

Appendix B: Photonic lattice fabrication

Fig. S2 shows the schematic of the photonic lattice's four primary fabrication processes. The fabrication process begins with the initial preparation of commercial Si wafers. These wafers are first coated with a 3.3 μm thick layer of SiO_2 as shown in **(a)**, which serves as an insulating layer. Following this, a 250 nm layer of Si_3N_4 is deposited onto

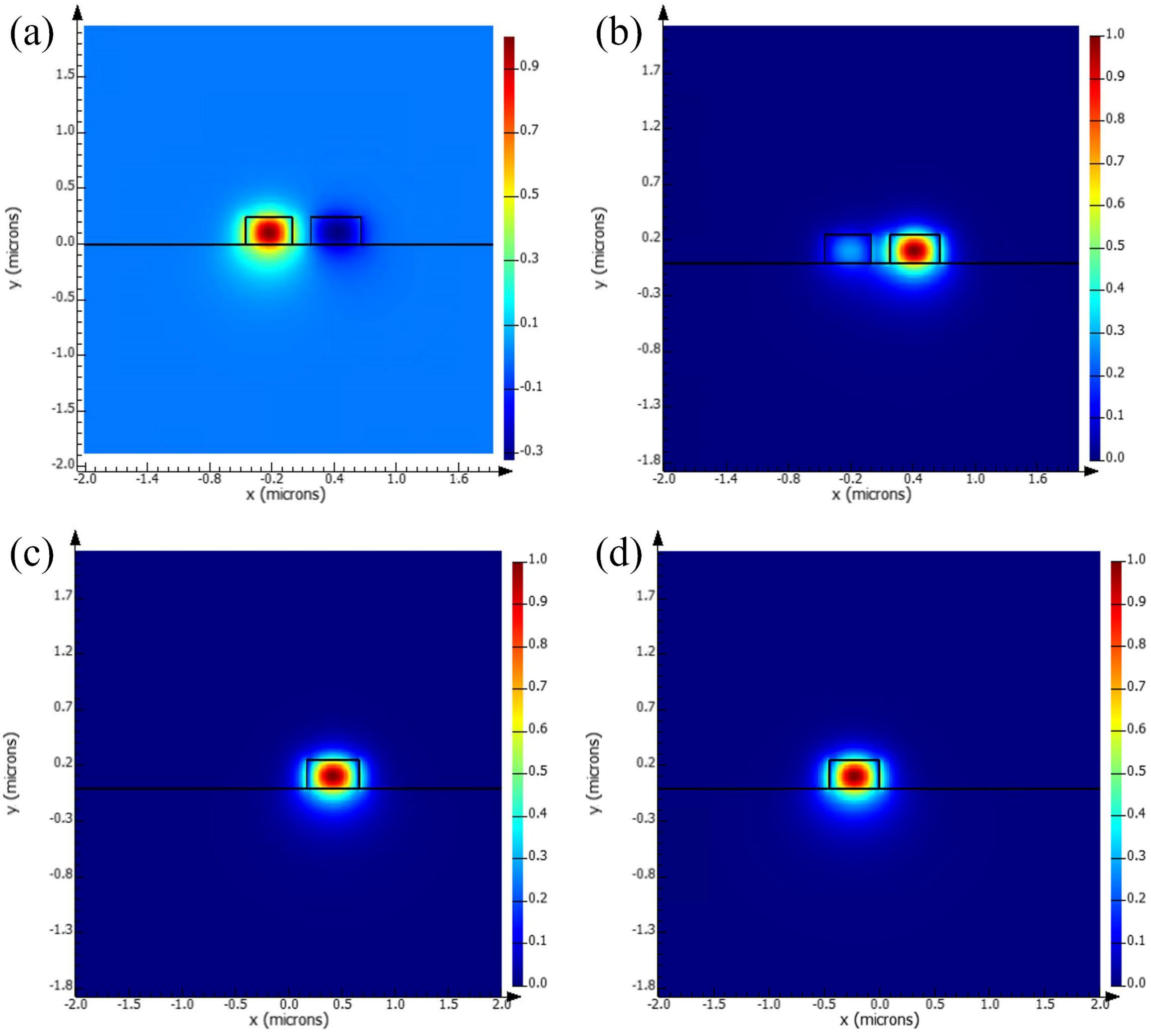

FIG. S3. The Lumerical simulation for the derivation of waveguides parameters

the SiO_2 layer, serving as the waveguide material. The waveguide structures are subsequently defined on the Si_3N_4 layer through the utilization of electron beam lithography. This process entails applying a negative-tone resist onto the Si_3N_4 layer, exposing it to an electron beam, and subsequently developing the resist using a developer solution. The unexposed regions are removed, leaving behind a precisely patterned resist layer as shown in **(b)**, which defines the waveguide structures.

Subsequently, the patterned resist layer is employed as a mask for dry etching of the Si_3N_4 layer as shown in **(c)**. This dry etching process utilizes a CF_4-based reactive ion etching technique, which selectively eliminates the Si_3N_4 material in the exposed regions defined by the resist layer. The etching process is precisely controlled to achieve the desired dimensions and shape of the waveguide structures.

Following the formation of the waveguide structures, the samples are cleaved to facilitate optical coupling with the Si_3N_4 waveguide side facets. Cleaving involves carefully cutting the sample along a specific axis, exposing the side facets of the waveguides. This step is crucial for enabling efficient coupling of light into and out of the waveguide structures. Finally, the remaining electron-beam resist is removed using a resist remover solution as shown in **(d)**, and the samples undergo a thorough cleaning and drying process to ensure their optimal condition for subsequent experiments and characterization.

Appendix C: Waveguides parameters design

To induce the on-site potential at specific dimers, it is necessary to finely tune the width of the corresponding waveguides while maintaining a consistent intra-cell and inter-cell coupling strength of 0.1 and 0.06. The on-site potential for each dimer can be depicted by Eq. S1

$$\beta_\lambda = \frac{2\pi}{\lambda} n_{eff}(\lambda) \tag{S1}$$

By following the methodology established in the reference [S1], we can ascertain the optimal widths of the waveguides and the appropriate gap distance between them. This procedure enables us to achieve precise control over the geometric parameters of the waveguide structure, ensuring the desired optical characteristics and facilitating the realization of specific on-site potentials within the system. Through the utilization of the Lumerical simulation methodology, we can obtain the isolated propagation constants of each waveguide within the dimer, denoted as β_1 and β_2. Additionally, we can determine the propagation constants of the dimer's even and odd modes, represented as β^+ and β^-. By these values, we obtain the difference as

$$\begin{cases} \Delta\beta = \frac{\beta_1 - \beta_2}{2} \\ \Delta\beta_{coup} = \frac{\beta^+ - \beta^-}{2} \end{cases} \tag{S2}$$

The coupling strength between these two asymmetric waveguides can be characterized as follows:

$$J = \sqrt{(\Delta\beta_{coup})^2 - (\Delta\beta)^2} \tag{S3}$$

The schematic of these simulations is shown in Fig. S3. From **(a)** and **(b)** we derive the β^+ and β^- by simulating the even and odd mode of the two waveguides system. In panels **(c)** and **(d)**, isolated waveguide mode simulations are performed, yielding the propagation constants β_1 and β_2, respectively.

Appendix D: Explicit estimates of the uncertainties

From scanning electron microscope images, we estimate uncertainties in the dimensions in the order of ± 10 nm (δr = 10 nm). According to the evanescent wave theory, the coupling t can be described by $t = A * exp(-k * r)$. By differentiating both sides of the equation, we get:

$$\delta t = |A * k \cdot exp(-k * r)|\delta r = k \cdot |t|\delta r \tag{S1}$$

In our waveguides platform, $k = 1/126.06$ nm and $\delta r = 10\ nm$, so the uncertainty of t is $\delta t \sim 0.08\ t$. By the same principle, the uncertainty of dimerization parameter Δ is also 0.08 Δ. For on-site potential, we have an empirical formula determined by the waveguide width w (notice δw also equals 10 nm):

$$\epsilon = A + B * w + C * w^2 + D * w^3 \tag{S2}$$

By differentiating both the left and right sides we get:

$$\delta\epsilon = B * \delta w + 2C * w * \delta w + 3D * w^2 * \delta w \tag{S3}$$

Substituting the values of B, C, D and the average width $w_a = 500nm$ into it can be calculated: $\delta\epsilon \sim 0.014$.

Appendix E: Analytical explanation of the reentrant-localization phase diagram

In this section, we focus on the physical reasons for the reentrant localization in the SSH model with the bimodal disordered potential on dimers, see Eqs. (1-2) in the main text. For this purpose, we, first, consider the limit of $\Delta = 1$, when the dimers are disconnected from each other, and then go away from this limit.

In the limit $\Delta = 1$, the system separates into a disjoint set of dimers of two types: with zero or finite on-site potential ϵ_n. This on-site potential leads only to the overall shift in the energy of the dimer levels. The coupling

$t(1+\Delta)$ between the sites within each dimer makes the energies of two eigenlevels separated by $2t(1+\Delta)$ with the energies

$$E_{\nu,\pm} = \nu\epsilon \pm t(1+\Delta) \ , \quad \nu = 0, 1 \tag{S1}$$

of the symmetric $\phi^i_{2n-1} = \phi^i_{2n}$ for $+$ and antisymmetric $\phi^i_{2n-1} = -\phi^i_{2n}$ for $-$ dimer wave-functions. These levels form the set of 4 degenerate sets of levels, which split at $\Delta \neq 1$ into the 4 bands, shown in Fig. 1(b) of the main text. The reentrant delocalization appears at $\Delta \neq 1$ when the spectral gap closes at

$$\epsilon \simeq 0 \text{ or } \epsilon \simeq 2t(1+\Delta) \ . \tag{S2}$$

Indeed, at $\epsilon \ll 1$ the energies of the symmetric $E_{\nu,+}$ dimer wave functions (and the antisymmetric ones $E_{\nu,-}$) appear to be in resonance with each other in pairs $\nu = 0, 1$. At the same time at $\epsilon \simeq 2t(1+\Delta)$ the energies $E_{0,+}$ of the symmetric dimer wave-functions with $\epsilon_n = 0$ appear to be in resonance with those $E_{1,-}$ of the antisymmetric ones with $\epsilon_n = \epsilon > 0$. Note that the same happens with $E_{0,-}$ and $E_{1,+}$ for $\epsilon \simeq -2t(1+\Delta) < 0$. As soon as the disorder is bimodal ($\epsilon_n = 0$ or ϵ), all the dimer wave functions of the bands $E_{\nu,+}$ or $E_{\nu,-}$ for $\epsilon = 0$ and $E_{0,+}$ and $E_{1,-}$ for $\epsilon = 2t(1+\Delta)$ are in resonance simultaneously. This leads to the transport across the system for such parameters and, thus, it is these resonances that delocalize the wave functions between resonance dimers in 2 intersecting band.

At $\Delta \neq 1$ for any ϵ far away from the resonance $|\epsilon|$, $|\epsilon \mp 2t(1+\Delta)| \not\ll t(1-\Delta)$ the degeneracy of each of the above 4 bands $E_{\nu,\pm}$ is lifted. Indeed, for any adjacent dimers with the same ϵ_n, the wave functions form the band of the width $2t(1-\Delta)$ around $E_{\nu,\pm}$. Due to the presence of disorder of the amplitude $\bar{\epsilon} = \min(\epsilon, |\epsilon - 2t(1+\Delta)|)$ between the sets of dimers with different ϵ_n, the states are exponentially localized, $|\phi^i_n|^2 \sim \exp\left[-|n-n_i|/\xi\right]$, with respect to some random localization centers n_i, with the localization length [S2]

$$\xi \simeq \begin{cases} 105.2\left[t(1-\Delta)/(2\bar{\epsilon})\right]^2 & \bar{\epsilon} \lesssim t(1-\Delta) \\ 1/\ln\left[\bar{\epsilon}/(et(1-\Delta))\right] & \bar{\epsilon} \gg t(1-\Delta) \ . \end{cases} \tag{S3}$$

Therefore all the states in the above 4 bands are localized.

However, when the gap between 2 bands closes (close to the resonance Eq. (S2)), the effective disorder ϵ in the localization-length expression (S3) for these 2 bands should be replaced by the corresponding difference $|\epsilon - 2t(1+\Delta)| \ll \epsilon$ for $E_{0,-}$ and $E_{1,+}$ and $|\epsilon + 2t(1+\Delta)| \ll \epsilon$ for $E_{0,+}$ and $E_{1,-}$. This leads first to the drastic increase of the localization length (S3) and as soon as ξ exceeds the number of sample sites N, the eigenstates in the corresponding 2 bands become delocalized. At the exact resonance $\epsilon = \pm 2t(1+\Delta)$, the Hamiltonian, projected to the corresponding intersecting bands is disorder-free with ballistic delocalized states.

All this explains the reentrant delocalization in the considered system close to the resonance condition (S2) with the reentrant localization at larger ϵ when no bands are in resonance again. This also makes clear why the gap closure in Fig. 1(b) matches well with the large value of the parameter $\eta = \log_{10}\left[\langle IPR\rangle \times \langle NPR\rangle\right]$ in Fig. 4 of the main text.

In the next two subsections we will consider the limiting cases of small $(1-\Delta) \ll 1$ in order to map the model, projected to the one or two subbands, to the 1d Anderson one. For this, we rewrite the initial eigenproblem

$$(E - \epsilon_n)\phi_{2n-1} = t(1+\Delta)\phi_{2n} + t(1-\Delta)\phi_{2n-2} \tag{S4}$$

$$(E - \epsilon_n)\phi_{2n} = t(1+\Delta)\phi_{2n-1} + t(1-\Delta)\phi_{2n+1} \tag{S5}$$

in terms of the symmetric $\phi_{S,n}$ and antisymmetric $\phi_{A,n}$ wave functions on the nth dimer of sites $2n-1$ and $2n$

$$\phi_{S/A,n} = \frac{\phi_{2n-1} \pm \phi_{2n}}{\sqrt{2}} \quad \Leftrightarrow \quad \phi_{2n-x} = \frac{\phi_{S,n} - (-1)^x \phi_{A,n}}{\sqrt{2}} \ , \quad x = 0, 1 \tag{S6}$$

as

$$\left[E - \epsilon_n - t(1+\Delta)\right]\phi_{S,n} = \frac{t(1-\Delta)}{2}\left[\phi_{S,n-1} + \phi_{S,n+1} - \phi_{A,n-1} + \phi_{A,n+1}\right] \tag{S7}$$

$$\left[E - \epsilon_n + t(1+\Delta)\right]\phi_{A,n} = -\frac{t(1-\Delta)}{2}\left[\phi_{A,n-1} + \phi_{A,n+1} - \phi_{S,n-1} + \phi_{S,n+1}\right] \ . \tag{S8}$$

In the cases $\epsilon \ll t(1-\Delta)$, Sec. E 1, and $|\epsilon - 2t(1+\Delta)| \ll t(1-\Delta)$, Sec. E 2, we consider the coupling of overlapping states on adjacent dimers $\phi_{S,n}$ with $\phi_{S,n-1}$ and $\phi_{A,n-1}$ respectively. Next, we consider the case of $(1-\Delta) \ll 1$ for simplicity.

1. Effective Hamiltonian close to the band crossing $\epsilon \ll t(1-\Delta)$

In this subsection, we consider the case of small $(1-\Delta) \ll 1$ and the vicinity of the band crossing at small disorder amplitude $\epsilon \ll t(1-\Delta)$. In this case, two different bands at $E \simeq \eta t(1+\Delta)$ are formed from the pairs of subbands $E_{\nu,\eta}$ with $\nu = 0, 1$. Let's consider only the symmetric one at $\eta = +1$ as the other one is given by the transformation $t \leftrightarrow -t$ and $\phi_{S,n} \leftrightarrow \phi_{A,n}$. Indeed, in the case of $e = E/[t(1+\Delta)] - 1 \ll 1$ in (S8) the small ratio $j = (1-\Delta)/(1+\Delta) \ll 1$ leads to the smallness of $\phi_{A,n}$ as

$$\phi_{A,n} \simeq \frac{j}{4+w_n} \left[\phi_{S,n+1} - \phi_{S,n-1}\right] \tag{S9}$$

where we have introduced $w_n \equiv \epsilon_n/[t(1+\Delta)] \ll j$. Substituting this to (S7), one obtains

$$[e - w_n]\, \phi_{S,n} = \frac{j}{2} \left[\phi_{S,n-1} + \phi_{S,n+1}\right] + O(j^2) \ . \tag{S10}$$

The latter term $O(j^2)$ leads in general to the higher-order distant hopping terms, decaying exponentially with a distance R as $H_{m,m+R} \simeq (j/4)^R \equiv e^{-R\ln(4/j)}$.

Equation (S10) maps to the 1d Anderson model with the rescaled bandwidth $e \simeq j, w \ll 1$ and the localization length (S3), as soon as it is smaller than the one above $\xi_0^{-1} = \ln(4/j) = \ln[4(1+\Delta)/)(1-\Delta)]$. In the current experimental setup with $\Delta = 0.25$ and $t = 0.08$, $\xi_0 \simeq 0.5$ which can be achieved only in the strong disorder limit of (S3), $\epsilon \simeq 10.5t(1+\Delta) \simeq 1 > 0.4$, beyond our experimental disorder amplitude.

Thus, the states in both subbands will look like delocalized as soon as the localization length (S3) is large compared to the system size N. In our experimental setup with $N = 100$, $t = 0.08$ and $\Delta = 0.25$ it leads to $\epsilon \simeq 0.3t(1-\Delta) \simeq 0.01$.

2. Effective Hamiltonian close to the band crossing $|\epsilon - 2t(1+\Delta)| \ll t(1-\Delta)$

In this subsection, we consider the case of small $(1-\Delta) \ll 1$ and the vicinity of the band crossing at the disorder amplitude $|\epsilon - 2t(1+\Delta)| \ll t(1-\Delta)$. In this case, two different bands at $E \simeq t(1+\Delta)$ are formed from the subbands $E_{0,+}$ and $E_{1,-}$.

In the case of $e = \frac{E}{t(1+\Delta)} - 1 \ll 1$ and $w = \frac{\epsilon}{t(1+\Delta)} - 2 \ll 1$, the symmetric wave-function amplitudes at the disordered dimers ($\epsilon_n = \epsilon$), $\phi^d_{S,n}$, and the antisymmetric ones at the clean dimers ($\epsilon = 0$), $\phi^c_{A,n}$, are small. Indeed, from (S7) and (S8) one obtains

$$\phi^d_{S,n} = -\frac{j}{4+2w-2e} \left[\phi_{S,n-1} + \phi_{S,n+1} - \phi_{A,n-1} + \phi_{A,n+1}\right] \tag{S11}$$

$$\phi^c_{A,n} = -\frac{j}{6+2e} \left[\phi_{A,n-1} + \phi_{A,n+1} - \phi_{S,n-1} + \phi_{S,n+1}\right] \ . \tag{S12}$$

Here we do not specify whether the dimers, adjacent to the above ones, are clean or disordered.

Neglecting as in the previous case all the $\phi^d_{S,n}$ and $\phi^c_{A,n}$ for the rest ones we obtain

$$e\phi^c_{S,n} = \frac{j}{2} \left[\phi^c_{S,n-1} + \phi^c_{S,n+1} - \phi^d_{A,n-1} + \phi^d_{A,n+1}\right] \tag{S13}$$

$$[e - w]\, \phi^d_{A,n} = -\frac{j}{2} \left[\phi^d_{A,n-1} + \phi^d_{A,n+1} - \phi^c_{S,n-1} + \phi^c_{S,n+1}\right] \ , \tag{S14}$$

where the symmetric terms $\phi^c_{S,n\pm1}$ in r.h.s. are present only if the corresponding $n\pm1$th dimer is clean ($\epsilon_n = 0$) and the antisymmetric ones $\phi^d_{A,n\pm1}$ – if it is disordered ($\epsilon_n = \epsilon$).

Up to the corresponding sign of the hopping terms this model is also equivalent to the 1d Anderson model. This leads to the same phenomenology of the localization with the length scale (S3) with $\bar\epsilon = |\epsilon - 2t(1+\Delta)|$.

Explicitly, one can map Eqs. (S13) and (S14) to Eq. (S10) with the following transformation

$$\begin{aligned}
&\left(\ldots, \phi^c_{S,n}, \ldots, \phi^c_{S,n+k_1}, \ldots, \phi^d_{A,n+k_1+1}, \ldots, \phi^d_{A,n+k_2}, \ldots, \phi^c_{S,n+k_2+1}, \ldots, \phi^c_{S,n+k_3}, \ldots\right) \quad \Rightarrow \\
&\qquad \Big(\ldots, \phi^c_{S,n}, \ldots, \phi^c_{S,n+k_1}, \ldots, \phi^d_{A,n+k_1+1}, -\phi^d_{A,n+k_1+2} \ldots, (-1)^{k_2-k_1-1}\phi^d_{A,n+k_2}, \ldots, \\
&\qquad\qquad (-1)^{k_2-k_1}\phi^c_{S,n+k_2+1}, (-1)^{k_2-k_1}\phi^c_{S,n+k_2+2}, \ldots, (-1)^{k_2-k_1}\phi^c_{S,n+k_3}, \ldots\Big) \ .
\end{aligned} \tag{S15}$$

Here we make $\phi^d_{A.n}$ sign-alternating and add the alternation to the link $\phi^d_{A,n} \leftrightarrow \phi^c_{S,n+1}$, but not to $\phi^c_{S,n} \leftrightarrow \phi^d_{A,n+1}$.

Thus, the mid-subband states will look delocalized as soon as the localization length (S3) is large compared to the system size N. In our experimental setup with $N = 100$, $t = 0.08$ and $\Delta = 0.25$ it leads to $|\epsilon - 2t(1+\Delta)| \simeq 0.3t(1-\Delta) \simeq 0.01$.

For the top disordered band, $e_t = \frac{E}{t(1+\Delta)} - 3 \ll 1$ (as well as for the bottom clean one, $e_b = \frac{E}{t(q+\Delta)} + 1 \ll 1$) the localization is guaranteed by the energy gapped states on clean (disordered) domains.

As on average half of the dimer are clean (disordered), the corresponding localization length for the top (ξ_t) and the bottom (ξ_b) subbands is given by the following expression

$$e^{-R/\xi_{t,b}} = \left(\frac{t(1-\Delta)}{2E_g}\right)^{R/2} \quad \Leftrightarrow \quad \xi^{-1}_{t,b} \simeq \frac{1}{2}\ln\left(\frac{2E_g}{t(1-\Delta)}\right) \, . \tag{S16}$$

The gap here is given by $E_g = \min(\epsilon, 2t(1+\Delta))$.

[S1] S. Nevlacsil, M. Eggeling, P. Muellner, G. Koppitsch, M. Sagmeister, J. Kraft, and R. Hainberger, roadband sin asymmetric directional coupler for 840 nm operation, *OSA Continuum* **1**, 1324 (2018).

[S2] F. M .Izrailev, S. Ruffo, and L. Tessieri, Classical representation of the one-dimensional Anderson model, *J. Phys. A: Math. Gen.*, **31** 5263 (1998).
